# The Effect of Diet on the Cardiac Circadian Clock in Mice: A Systematic Review

**DOI:** 10.3390/metabo12121273

**Published:** 2022-12-15

**Authors:** Ana Beatriz Rezende Paula, Letícia Teresinha Resende, Isabela Alcântara Barretto Araújo Jardim, Bianca Iara Campos Coelho, Denise de Coutinho Miranda, Alexandre Martins Oliveira Portes, Maria Cecília Teles, Ana Maria de Lauro Castrucci, Mauro César Isoldi

**Affiliations:** 1 Laboratory of Cell Signaling, Research Center in Biological Science, Institute of Exact and Biological Sciences, Federal University of Ouro Preto, R. Quatro, 786, Bauxita, Ouro Preto 35400-000, Brazil; 2 Laboratory of Comparative Physiology of Pigmentation, Department of Physiology, Institute of Biosciences, University of São Paulo, R. do Matão, Trav. 14, No. 101, São Paulo 05508-090, Brazil

**Keywords:** circadian rhythms, diet, heart, metabolism, restricted feeding

## Abstract

Circadian rhythms play important roles in regulating physiological and behavioral processes. These are adjusted by environmental cues, such as diet, which acts by synchronizing or attenuating the circadian rhythms of peripheral clocks, such as the liver, intestine, pancreas, white and brown adipose tissue, lungs, kidneys, as well as the heart. Some studies point to the influence of diet composition, feeding timing, and dietary restriction on metabolic homeostasis and circadian rhythms at various levels. Therefore, this systematic review aimed to discuss studies addressing the effect of diet on the heart clock in animal models and, additionally, the chronodisruption of the clock and its relation to the development of cardiovascular disorders in the last 15 years. A search was conducted in the PubMed, Scopus, and Embase databases. The PRISMA guide was used to construct the article. Nineteen studies met all inclusion and exclusion criteria. In summary, these studies have linked the circadian clock to cardiovascular health and suggested that maintaining a robust circadian system may reduce the risks of cardiometabolic and cardiovascular diseases. The effect of time-of-day-dependent eating on the modulation of circadian rhythms of the cardiac clock and energy homeostasis is notable, among its deleterious effects predominantly in the sleep (light) phase and/or at the end of the active phase.

## 1. Introduction

The rotation of the Earth on its axis is marked by a light phase and a dark phase, both with distinct temperature and radiation conditions. This light/dark cycle (LD) is interpreted through the circadian system, represented by a central clock located in the suprachiasmatic nucleus (SCN) of the hypothalamus, and peripheral clocks distributed in other regions of the brain and peripheral organs [1,2,3]. Each nucleated cell has a clock, which adjusts itself through external or internal clues also called *zeitgebers* (ZT).

The central clock is adjusted daily by light, the main environmental cue. In this way, the central clock sends signals to the peripheral clocks, thus synchronizing their circadian rhythms [4,5]. The cellular clock machinery is represented by transcriptional, translational, and post-translational events, loops of positive and negative feedback, performed by a set of genes. The genes encoding the clock mechanism include *Clock* and *Bmal1* (positive loop) and *Per1/2/3* and *Cry1/2* (negative loop) [5].

In mammals, the circadian clock influences practically all physiological and behavioral aspects, such as the sleep–wake cycle, body temperature, energy metabolism, and the physiology of various organs. [6]. Using the heart as an example, about 6 to 13% of its transcriptome can be controlled by the clock [7,8].

Considering peripheral oscillators, the role of external cues in modulating circadian rhythms is already well described. Food is considered an important synchronizer for peripheral clocks [9,10], as well as metabolism, which has an important effect on both central and peripheral clocks [11]

Thus, restricted feeding (RF) to a certain period profoundly affects the physiology and behavior of animals. Among these changes, one may mention changes in locomotor activity, body temperature, food anticipatory behavior, and hormonal secretions [12]. Studies on rodents have shown the beneficial effects of RF on metabolic pathways [13,14].

Mice fed control or high-fat diet (HFD) by RF only in the dark phase showed increased fatty acid oxidation in cardiac muscle, stimulation of fatty acid-responsive genes, improvement of myocardial contractile function, and no alteration in cardiac hypertrophic. RF in wakefulness resulted in metabolic flexibility for cardiac lipid metabolism [15].

Thus, it is important to investigate the relationship between diet and the circadian clock. Changes in the functioning of the clock by environmental signals, especially food, can lead to a better or worse functional picture of physiological and behavioral processes.

In light of the above, this systematic review aims to discuss studies that address the effect of diet on the heart clock in mouse models and the chronorupture of the clock and its relationship to the development of cardiovascular disorders in the last 15 years.

## 2. Materials and Methods

### 2.1. Search Strategy and Data Extraction

The PRISMA guide [16] was used to construct the systematic review. For the design of this study, a search was conducted in the electronic databases: PubMed (https://pubmed.ncbi.nlm.nih.gov/ accessed on 3 August 2022), Scopus (https://www-scopus.ez28.periodicos.capes.gov.br/search/form.uri?display=basic&zone=header&origin=#basic accessed on 3 August 2022) and Embase (https://www.embase.com/landing?status=grey) accessed on 3 August 2022. The research was conducted during the entire month of August 2022. The search of the articles in the bases had the combination of the following key words:

- For PUBMED: (“diet” OR “nutrition” OR “feeding”) AND (“circadian clock” OR “chronobiology” OR “biological clock” OR “clock proteins” OR “clock gene”) AND (“heart” OR “cardiac” OR “cardiomyocyte” OR “ventricle”) AND (“mice” OR “mouse”);
- For Scopus: (“diet” OR “nutrition” OR “feeding”) AND (“circadian clock” OR “chronobiology” OR “biological clock” OR “clock proteins” OR “clock gene”) AND (“heart” OR “cardiac” OR “cardiomyocyte” OR “ventricle”) AND (“mice” OR “mouse”);
- For Embase: (“diet” OR “nutrition” OR “feeding”) AND (“circadian clock” OR “chronobiology” OR “biological clock” OR “clock proteins” OR “clock gene”) AND (“heart” OR “cardiac” OR “cardiomyocyte” OR “ventricle”) AND (“mice” OR “mouse”).

For the purpose of this study, the most current publications, produced between 2007 and 2022, were considered. This was considered important, considering the evolution of studies in the area. The database search was performed by two researchers: A.B.R.P. and L.T.R. Both researchers read and selected the articles. In cases of conflict, the articles were reevaluated by the same two researchers, A.B.R.P. and L.T.R.

**Figure 1:**
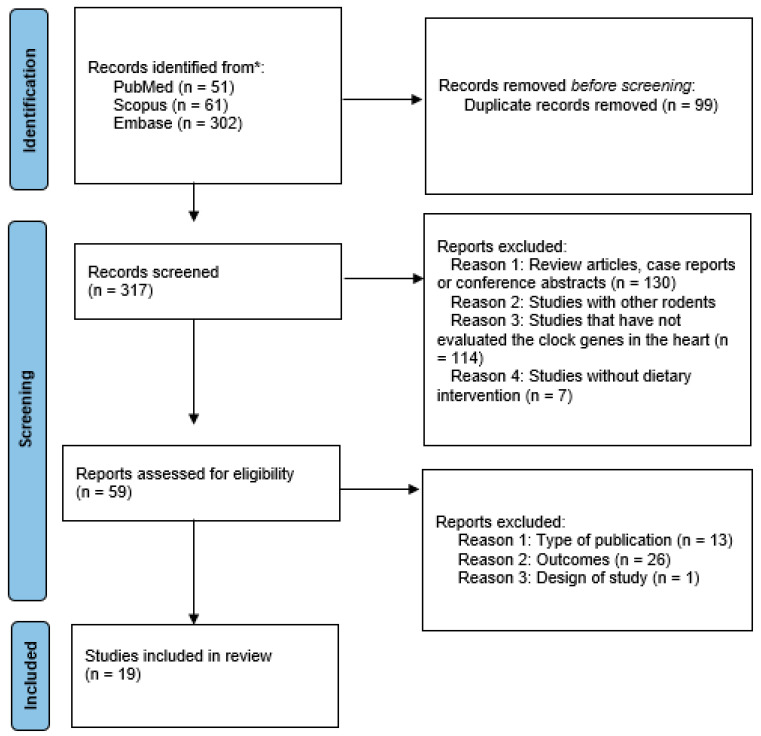
Flow diagram with search strategy.

Duplicates were removed before screening the articles retrieved. Before establishing the first selection, the pre-defined criteria according to PICOS (“Population”, “Intervention”, “Comparison”, “Results”, and “Study Design”) were adopted as inclusion criteria to elect the articles (Table 1). The online software Rayyan (https://www.rayyan.ai/ accessed on 3 August 2022) was used to select the articles. A detailed search flowchart (Figure 1) illustrates articles that have been shown to meet the established eligibility criteria. The study protocol was registered in the international database PROSPERO with the reference CRD42022360982.

After reading the articles, the information was recorded in a catalog file for each study, which contained the following data: title, publication year, population, study objective, methodology, intervention, results, and conclusion. Only the most relevant data for the construction of this article were cataloged; from this summary, data were used in order to synthesize the results.

### 2.2. Quality Assessment of Studies

The risk of bias (RoB) was assessed for each study by “SYRCLE’s RoB tool” [17]. This tool was based on the Cochrane Collaboration RoB Tool and it aims to assess the methodological quality in animal experiments. The quality assessment was performed by two researchers and disagreements were resolved between them.

## 3. Results

### 3.1. Literature Data

The database search returned 414 articles, 51 from PubMed, 61 from Scopus, and 302 from Embase. Duplicate articles removed prior to screening were 99, leaving 317. Inclusion and exclusion criteria were applied according to PICOS. A total of 59 articles were selected, of which 40 were excluded: publication type, results, and study design. Thus, 19 studies that met all inclusion and exclusion criteria were contemplated (Figure 1).

Of these 19 studies, 6 were RF studies [18,19,20,21,22,23], 7 involved HFD [24,25,26,27,28,29,30,31], 2 were ketogenic diets [25,32], and 4 were other types of dietary intervention [33,34,35,36], according to Table S1. Table 2 details information about the objectives and methodologies of the selected studies. The main results about the circadian clock in the heart and other tissues are presented in Table 3. Table 4 points out the metabolic changes observed in the studies.

### 3.2. Quality Assessment of Studies

All the included studies in the systematic review had inappropriate random sequence generation. Overall, the studies presented a high risk for selection bias (random sequence generation, baseline characteristics, and allocation concealment), performance bias (random housing and blinding of participants and personnel), and detection bias (random outcome assessment and blinding of outcome assessment). Attrition bias (incomplete outcome data) was intermediate. Reporting bias (selective reporting) was low. The results of the risk of bias in the included studies are shown in Figure S1.

## 4. Discussion

Light is an important environmental cue in dragging biological rhythms. However, other cues are capable of adjusting circadian rhythms, such as diet. Some studies point to the influence of diet composition, meal timing, and dietary restriction on metabolic homeostasis and circadian rhythms at various levels. Understanding the mechanisms underlying these influences will likely provide important insights into the pathogenesis of diet-associated cardiometabolic disorders.

Feeding is a strong synchronizer for the peripheral clocks, as is the timing of the day feeding. Given this, we sought to include in this review studies that evaluated the effects of restriction feeding (RF) on clock genes. RF is characterized by a limited period and duration of food access without caloric reduction. Some studies have evaluated the effects of dietary restriction on the expression of clock genes [17,18,24,27,33]. Bray et al. [20] sought to understand the effect of RF in the dark phase in male C57Bl/6J mice. The authors observed markedly different diurnal variations among those animals that consumed the diet in the dark phase compared to those in the light phase. The animals fed in the light phase consumed a larger amount of food immediately after accessing the diet, whereas those in the dark phase did not show the same response. The authors observed that the heart’s circadian clock genes did not change phase, but the amplitude of oscillations of clock gene expression was often decreased in this organ. Such findings were observed in adipose tissue and skeletal muscle as well, whereas, in the liver, there was a phase shift of circadian clock genes in those mice fed in the light phase. These results suggest that entrainment-induced feeding is more robust in the liver, and RF in the light phase led to a desynchronization between metabolically active tissues. Furthermore, the authors point out that RF, in the dark phase, resulted in increased caloric intake, reduced energy expenditure, and dependence on fatty acid oxidation. Additionally, Goh et al. [18], when investigating the effects of RF on clock genes, found that access to dietary restriction led to phase shifting in the peripheral clocks of wild-type animals. In this same assay, using PPARα-deficient mice, they observed significant modulation in the heart muscle clock, shifting the acrophase of circadian gene expression by up to an additional 8 h. They observed that dietary restriction reduced the amplitude of expression of *Bmal1* and *Rev*-*Erbα* genes in WT and homozygous *Pparα*-null (*Ppar*α-null) mice, respectively, and increased *Bmal1* in *Pparα*-null mice, whereas, under *ad libitum* conditions, the amplitude and acrophase of clock genes were similar in both genotypes. These knockout animals showed altered cardiac metabolism using glucose as an energy source, leading to loss of contractile function and decreased energy reserves. Under prolonged fasting conditions, these animals developed hypothermia, due to the altered brown adipose tissue (BAT). These findings may be linked to the current observation that BAT and cardiac tissue from *Ppar*α null mice showed altered circadian food transcription factor gene expression.

Reilly et al. [19] investigated the possibility of catecholamines to modulate the rhythmicity of peripheral clocks in dopamine β-hydroxylase knockout mouse models (*Dbh*^−/−^) under RF during the light phase. This model is characterized by the non-expression of the enzyme dopamine B-hydroxylase, which takes part in catecholamine biosynthesis [37]. They observed that endogenous concentrations of the catecholamines, norepinephrine, and epinephrine, exerted no effect on the function of peripheral circadian clocks *in vivo,* among them, the heart. On the contrary, feeding time was shown to be an important modulator of peripheral circadian oscillators.

Despite this, they did not study the effect of the RF; Noyan et al. [22] worked with short-term caloric restriction (CR) (30% less of total calories) and investigated its protective effect on ischemic mouse hearts. CR caused a change in gene expression. Bioinformatics analyses showed enriched pathways associated with antioxidant processes, circadian rhythms, and the biological clock. Moreover, short-term CR resulted in increased expression of the clock genes, *Per1* and *Per2*. Studies point to the involvement of clock genes in physiological processes such as energy balance, coordinating and modulating energy metabolism, transcription, signaling, and contractile functions in the heart [38]. In view of this, heart clock genes may be associated with the beneficial effects of calorie restriction [39]. Further, studies point to the protective effect of the *Per2* gene in myocardial ischemia [40]. Therefore, cardiac clock genes may be involved in mechanisms protecting the heart against ischemia associated with short-term CR by adapting cellular metabolism.

Mukherji et al. [21], however, sought to understand the effect of switching feed from the active to the resting phase (RF condition) on peripheral clock genes. After 8 days of RF in the resting phase, transcript analyses of cardiac clock components showed that the clock genes *Per1*, *Per2,* and *Rev-Erbα* were affected by this condition. RF led to an extra production of corticosterone. Notwithstanding, under *ad libitum* conditions, endogenous corticosterone did not affect *Rev-Erbα* expression in the heart, since they were at basal levels. Subsequently, in the 4 days of RF, there was a delay in *Rev*-*Erbα* expression and, consequently, a delay in peripheral clock shift. Administration of the glucocorticoid antagonist RU486 or adrenalectomy led to early activation of PPARα in the heart, consequently, stimulating *Rev*-*Erbα* expression. The authors concluded that the change in clock gene components under RF conditions is associated with metabolic reprogramming that directly affects circadian clock expression. In the study by Xin et al. [23], they evaluated the effects of reverse feeding in peripheral tissues on metabolism and circadian physiology in mice, among them, the heart. The diurnal rhythms of the clock genes in the heart exhibited a phase shift from 0–3 h. On the other hand, when comparing male and female mice, the diurnal rhythms were damped by RF in male mice. That is, there is a sex-related difference in clock drag under RF. Even though the authors observed a greater resilience in phase drag by reverse feeding in the heart transcriptome, cardiac diurnal metabolites were dragged within one week. In addition, removing the timing signals from the SCN by exposing the animals to constant light (LL) cycle-facilitated phase drag by feeding in both the heart and the other tissues except the liver. Analyses of the cardiac metabolome showed that reversed feeding dragged the diurnal rhythms of fatty acid oxidation and acylcarnitine metabolites. These findings show that cardiac metabolism is influenced by feeding cycles and the circadian clock.

Some studies evaluated the effects of HFD in WT mice [25,28,32] or knockout models, such as *ApoE*^−/−^ mice [24], *Clock* ^Δ19/Δ19^ mice [30], and cardiomyocyte clock mutant (CCM) mice [27], as well as a transgenic model that overexpresses PER2 [26]. Hou et al. [24] investigated the effect of hyperlipidemia induced by an HFD on the expression of clock genes in *ApoE*-deficient mouse model of atherosclerosis (*ApoE*^−/−^). The authors observed that diet affected the peripheral circadian clocks, but had no effects on the central clock. In the cardiac circadian clock, the peak mRNA levels of the clock genes, *Bmal1*, *Per2*, and *Cry1*, showed a four-hour delay in the onset of the subjective dark period in knockout animals, independent of diet, reinforcing that apolipoprotein E (ApoE) is involved in the expression of these genes. The same phenomenon was observed for plasma lipid levels, having a peak at the onset of the dark period, CT12, in *ApoE*^−/−^ mice fed with HFD, with no variation in serum levels for WT mice. The authors point out that these variations in serum lipid levels possibly affected the expression of circadian clock genes, mediated by the transcription factors PPARα, retinoid X alpha receptor (RXRα), and REV-ERBα. This circadian disruption associated with the absence of ApoE may be related to the development of atherosclerotic processes and some acute cardiovascular diseases, as pointed out by more recent studies [41,42,43].

Mia et al. [31] sought to evaluate the effect of HFD diet-induced obesity on cardiac metabolic flexibility. Of note, metabolic flexibility is characterized by the adaptation to metabolic and energetic changes in response to physiological stimuli. The authors observed that HFD-induced obesity resulted in increased body weight and adiposity, and altered the 24-h rhythms of body substrate selection during the LD cycle. However, the oxidation of glucose and/or fatty acids by cardiomyocytes during the LD cycle was preserved. That is, metabolic flexibility is preserved for cardiac substrate oxidation in high-fat-fed mice. This diet also stimulated markers related to cardiac hypertrophy, cardiac fibrosis, and steatosis. RNA-sequencing (RNAseq) analyses evidenced diurnal changes in the cardiac transcriptome, particularly, in metabolism-related genes, with only 22% of transcripts unaffected by HFD. Importantly, of the transcripts that were not affected, the clock genes, *Bmal1*, *Clock*, *Npas2*, *Nr1d1*, *Nr1d2*, *Per2*, *Per3*, and *Cry2* stand out. According to the authors, there is the possibility that the heart clock orchestrates the persistent day-night differences in cardiac oxidative metabolism during obesity. In lipid metabolism, triglyceride synthesis was impaired by obesity, which was associated with attenuation in day-night fluctuations of cardiac lipidome. At another time, the authors investigated the effect of RF with HFD in the dark phase only. RF restored the changes in lipid metabolism and cardiac remodeling. Given this, a detrimental effect of HFD-induced obesity on the metabolic flexibility of lipid metabolism is evidenced, and it is, in turn, partially reversed by modulating the timing of food intake. Reitz et al. [30], meanwhile, evaluated whether HFD-fed Clock^Δ19/19^ mice develop cardiovascular disease since this diet, in this model, results in metabolic syndrome and obesity, risk factors for cardiovascular disorders. In spite of the fact that the animals exhibited a cardiometabolic risk profile, surprisingly, they did not develop cardiac dysfunction and showed preserved cardiac structure and function. Microarray and bioinformatics analyses revealed a pattern of antioxidant activity associated with increased serum levels of the enzymes cardiac catalase (CAT) and glutathione peroxidase (GPx) and the *Ppary* gene expression, and reduced activation of oxidative stress-related pathways. These findings demonstrate the important role of circadian mechanisms in mediating resilience to cardiovascular disease outcomes. These studies [31,32] only reinforce the influence of the clock on metabolic homeostasis, since, under a condition of circadian chronorupture, there is impaired cardiac metabolism, but the cardiac function is maintained. Studies point out that even under conditions of a non-intact circadian mechanism, cardiac remodeling can be enhanced. However, it is important to note that circadian mechanisms are important since the genes and proteins involved in the observed outcomes are under circadian transcriptional control. Tsai et al. [27] studied the role of the cardiac circadian clock in cardiac metabolic adaptation under HFD for 16 weeks. For this, the authors used a mouse model, in which, CLOCK protein expression is selectively impaired, termed the cardiomyocyte clock mutant (CCM) mice. The animals showed an altered myocardial response to HFD, as well as altered diurnal rhythms of triglyceride and fatty acid metabolism. The diurnal rhythms of myocardial triglyceride levels were markedly attenuated in the heart of the CCM mice, which was associated with circadian mechanisms mediating the regulation of lipolysis over synthesis. Nonetheless, when subjected to an RF condition, the heart lipid metabolism responded differently. Feeding at the end of the active phase resulted in myocardial steatosis with a greater propensity for triglyceride synthesis.

Wang et al. [28] evaluated the effects of HDF-induced maternal obesity on clock and metabolism genes in the liver and heart of their offspring. In this study, they evaluated the pups at two time points, pups with 17 days of age (P17) and pups with 35 days of age (P35). The authors observed that HFD during pregnancy and lactation strongly impacted the expression of clock genes, metabolism genes, and inflammatory pathways in both the heart and liver. In the heart, *Bmal1* and *Per2* genes showed a robust oscillation compared to the metabolism genes carnitine palmitoyltransferase 1b (*Cpt1b*) and *Pparα*, which was associated with the early developmental stage of HFD pups. That is, the phase and amplitude changes were more expressive in the pups at 17 days postnatal of HFD-fed female mice. The expression of genes in inflammatory processes was higher in P17 proles from obese females. As for metabolism genes, there was a difference in the pattern of oscillation between the P17 and P35 groups. The authors associated the maternal cues during the first postnatal weeks, whereas, the more “mature” pups (P35) were already influenced by feeding, with peripheral clocks being adjusted by the feeding schedule, in addition to food intake being an important environmental cue for dragging circadian rhythms of peripheral clock genes [9]. Furthermore, diet composition and the prenatal microenvironment would strongly affect the oscillation pattern of circadian rhythms in the heart [28].

Oishi and colleagues have developed studies involving the combination of a high-fat, high-sucrose diet [19,28] on fibrinolysis [19] and on the effect of endogenous insulin [29] at two time points. In the study [26], Oishi et al. [19] evaluated the role of PER2 in plasminogen activator inhibitor-1 (PAI-1) gene expression in a transgenic mouse model overexpressing *Per2* (*Per2*/Tg) and WT mice, both with and without obesity induced by a high-fat/high-sucrose diet (HFSD). HFDS-fed animals showed body weight gain, hypercholesterolemia, and hyperinsulinemia and developed insulin resistance in both genotypes, but did not have their plasma triglyceride levels increased. However, these findings did not differ significantly between the groups. Therefore, the authors suggest that the PER2 protein has no involvement in the metabolic regulation of obese animals induced by an HFSD diet. The PAI-1 gene was suppressed in the heart of *Per2*/Tg animals, regardless of diet type. This was associated with higher cardiac *Per2* gene expression since no PAI-1 gene suppression and no change in *Per2* mRNA levels was observed in adipose tissue and liver. However, the expression levels of *Bmal1* mRNA were not altered in the heart of *Per2*/Tg mice, but its attenuation was observed in the liver and adipose tissue. Since PAI-1 levels oscillate over the 24-h rhythm in various organs, such as the liver, adipose tissue, and heart [44], the findings of this study suggest that components of the circadian machinery are strongly involved in regulating its expression in the heart. In summary, diet-induced obesity increases PAI-1 levels, but its transcription is suppressed by the *Per2* gene. In 2017, Oishi et al. [29] conducted interventions with the same diet, HFSD. In this study, the authors investigated the effect of endogenous insulin dependent in the feeding cycle on the regulation of peripheral clocks. The animals were fed either in the light phase or in the dark phase for one week. RF led to the synchronization of the circadian rhythms in the insulin of the hormones, glucagon-like peptide-1 (GLP-1), glucose-dependent insulinotropic polypeptide (GIP), and hyperinsulinemia in the light period. When analyzing the expression profile of the clock genes, *Per1*, *Per2,* and *Dbp*, no marked influence of light phase feeding on the heart clock was observed. Only the liver was affected concerning the circadian phase of expression. For that reason, the authors concluded that insulin and RF are not dominant ZT for some peripheral clocks, except for the liver. Nevertheless, the exogenous insulin was able to drag the peripheral clocks. Given these findings, humoral signals involved in synchronizing peripheral clocks are unlikely dominant ZT, since they are strongly influenced by some environmental cues, such as temperature, diet composition, meal timing, physical activity, and light [45]. For example, circulating insulin concentrations increase dramatically in response to diets. Therefore, humoral time signals, such as glucocorticoids and insulin, may serve to stabilize rather than phase-determine peripheral clocks in mammals [46].

Some studies [20,23] have assessed the effect of the ketogenic diet (KD), characterized by high concentrations of lipids and low carbohydrate and protein content [47]. Commonly used in weight loss and diabetes, little is known about its effect on cardiovascular health [48]. Oishi et al. [32] investigated the effect of KD on the temporal expression profile of PAI-1 and clock in peripheral tissues, liver, kidney, adipose tissue, and heart. KD led to hypoglycemia, increased FFA and ketone body levels, and a phase advance on clock genes, PAI-1 mRNA levels, and the rate of behavioral activity in *ad libitum*-fed mice. The mRNA expression acrophases of the *Per2* and *Dbp* genes were advanced by 5.6 and 6.0 h, respectively, in the heart. When transferred from LD to DD, the phase diet advanced the clocks that govern rhythmic behavior. The authors point out that KD exerted an effect similar to that of CR, fasting, and hypoglycemia, which was associated with cellular energy status. In another report, Oishi et al. [25] compared the effect of KD on the temporal expression profile of clock genes (*Bmal1* and *Rev*-*Erbα*) and clock-controlled genes (*Dbp*), and on the induction of PAI-1 gene expression in the liver and heart of PPARα-free mice. A phase advance in *Bmal1* expression levels and a decrease in mean *Dbp* gene expression level were observed in the heart of both genotypes. Similar to the 2009 study [32], Oishi et al. [25] again observed a phase-advancing effect on the rate of behavioral activity in KD-fed animals, independent of PPARα, when transferred from an LD to a constant darkness (DD) condition. They showed that PPARα does not influence the phase-advancing effect on the rate of expression in clock genes and peripheral behavioral activity. This phase change of the KD-induced circadian clock may be influenced by the cellular energy status, such as the ratio of reduced and oxidized nicotinamide adenosine dinucleotide energy [NAD(P)H/NAD(P)+] [49]. Energy homeostasis is maintained by AMP kinase (AMPK), which is activated by some factors such as CR, fasting, hypoglycemia, and KD [50]. Its activation induces PER2 degradation, activating casein kinase Iɛ, leading to a phase advancement of the circadian clock in vitro [50]. Thus, activated AMPK may be involved in mediating circadian rhythms induced by KD clock regulation in tissues.

Finally, we found reports on other dietary profiles, such as low/high-branched chain amino acids (BCAA) diet [35], high-fiber diet associated with acetate supplementation [33], biotin-rich diet [35], and low-phosphate diet [34]. Latimer et al. [36] observed that the time of day of BCAA intake influences cardiac parameters, which was associated with the circadian clock of the heart. WT mice that consumed a BCAA-rich meal at the end of the active phase (dark phase) revealed cardiac remodeling and influence on cardiometabolic parameters, with increased responsiveness of BCAA-induced mechanistic target of rapamycin (mTOR), the signaling pathway involved in cardiac hypertrophy [51]. However, cardiomyocyte-specific *Bmal1* knockout (CBK) lost these time-of-day-dependent differences in BCAA-induced activation of mTOR signaling. Components of BCAA metabolism, such as cardiac amino acid levels, ribosomal RNA, and DEP domain-containing mTOR-interacting protein (DEPTOR), a repressor of mTOR activation, showed significant 24-h cardiac clock-dependent oscillations in WT animals. In CBK animals, on the other hand, repression of BCAA catabolism genes was observed, leading to increased cardiac BCAA levels and, ultimately, an exacerbated activation of mTOR signaling and cardiac hypertrophy.

Macro- and micronutrient content and sources have a strong impact on health and the risk of developing chronic non-communicable diseases [52,53]. For example, fruit and vegetable consumption are related to a lower incidence of diabetes, hypertension, and metabolic syndrome. In the study by Marques et al. [33], the effect of a high-fiber diet and acetate supplementation on the gut microbiota, and the cross-talk between the kidney, liver, and heart in hypertensive mice were investigated. Dietary fiber and acetate positively modified the size and population diversity of the gut microbiome, and also reduced diastolic and systolic blood pressure and renal fibrosis, which was associated with the downregulation of a marker related to cardiovascular disease, renal fibrosis, and inflammation. The authors observed an upregulation of circadian clock genes in the kidney and heart. In other words, dietary fiber and acetate would be acting as ZT for the renal and cardiac circadian clocks, and these, possibly, may be involved in molecular pathways that culminated in improved cardiovascular health and function. However, further studies are needed on the molecular mechanisms involved.

Circadian clocks are important agents in modulating metabolism. Conversely, the mechanism involved is not well defined. The processes of transcription, translation, and post-translational modifications also have circadian regulation. Protein biotinylation is an important post-translational modification for protein activity [54]. Murata et al. [35] described the effect of dietary biotin intake on a brain and muscle ARNT-Like 1 (*Bmal*1)-BirA* knock-in (*Bmal*1-BioID) mouse model for protein biotinylation in various tissues. In order to build the model, the authors used a biotin ligase, BirA*. In addition, BMAL1 protein acts as a transcription factor in clock-controlled genes. Consequently, this mouse model was used to investigate the tissue-specific protein–protein interaction of BMAL1 protein. The biotin-rich diet induced protein biotinylation in the brain and in other tissues such as the heart.

In another study [34], the effect of a low-phosphate diet on the circadian clock of bone tissue and the heart was evaluated. That is, would a condition of hypophosphatemia affect circadian function? The authors observed that the expression levels of clock genes, in the tissues evaluated, were higher in the mice on a low phosphate diet. In the heart, there was phase advancement and significantly higher levels for the *Per1*, *Per2*, and *Per3* genes; the *Bmal1* gene expression peak shifted from ZT24 to ZT21. Therefore, a diet-induced hypophosphatemia condition results in phase shifting for the clock genes. In conjunction, 1.879 genes were associated with diet and showed a circadian expression pattern; among them, there were genes involved in hypoxia signaling in the cardiovascular system and cardiac morphology. Thus, it was suggested that circulating phosphate levels modulate the heart clock and control the circadian functions of the heart.

In summary, studies have linked the circadian clock to cardiovascular health and suggest that maintaining a robust circadian system may reduce the risks of cardiovascular and cardiometabolic diseases. Noteworthy is the effect of the time-of-day-dependent feeding on the modulation of the circadian rhythms of the heart clock and energy homeostasis, its deleterious effects predominantly occurring in the sleep (light) phase and/or at the end of the active phase. Another important point is the composition of the diet in macro- and/or micronutrients, such as fat, carbohydrates, protein, fiber, and minerals. Some of these nutrients may act on molecular and/or metabolic pathways by modulating the circadian clock of the heart and of the other peripheral organs. The circadian clock plays an important role in metabolic regulation, and a disrupted condition may contribute to the development of heart disorders.

## 5. Limitations and Future Research

As a limitation of the study, only the articles selected by the three databases were cataloged. Therefore, articles that were not included in the databases were not considered, even those articles that were cited in the selected papers. Moreover, there may be studies that have indirectly evaluated the heart, but due to the restriction of the keywords, they were not found in the databases. According to the articles evaluated, the effect of food restriction on the biological clock is notorious, and it can be harmful or beneficial, depending on the timing of the intervention. This may reflect in the experimental design and outcome of studies and may be another bias for research involving diet, clock, and metabolic parameters. More research is needed in the area in order to better understand the effects of various diets on the biological clock and metabolism.

## 6. Conclusions

Based on the findings of this systematic review, it highlights the important role of diet in modulating peripheral clocks, especially the heart, and cardiac metabolism. Furthermore, diet composition and feeding schedule (restricted feeding) can affect cardiac parameters and the expression of clock genes.

## Figures and Tables

**Table 1:** Inclusion and exclusion criteria used according to PICOS.

Parameters	Inclusion Criteria	Exclusion Criteria
Population	Studies conducted with mice	Studies conducted with other rodents
Intervention	Diet, Restricted feeding	No dietary intervention
Comparison	Not applicable	Not applicable
Results	Clock genes	Other genes
Study Design	Case-control studies and experimental trials Published after 2007	Literature review, cross-sectional studies, letters to the editor Published before 2007, studies not in the English language

**Table 2:** Objectives and methodologies used in the studies.

Author/Year	Species/Strain/Sex	Objectives	Diets/Composition	Dietetic Intervention	Methodology
Goh et al., 2007 [18]	Male C57Bl/6N × Sv/129, C57Bl/6N × Sv/129 homozygous *Pparα*-null mice.	To investigate the impact of PPARα deficiency on the expression of genes encoding circadian transcription factors and PPARα responsive proteins in peripheral tissues following temporally restricted food access.	Regular chow diet (Purina 5015)	Mice were compared under *ad libitum* or restricted food access for the expression of circadian transcription factor-encoding mRNAs.	Gene expression by qPCR
Reilly et al., 2008 [19]	Males *Dbh*^+/−^, *Dbh*^−/−^, C57Bl/6J mice	To investigate the role of the SCN on the entrainment of the heart and vasculature oscillators.	Regular chow diet or RF (for 6 h).	Mice were submitted to RF during the light period (7 a.m. to 7 p.m.) or *ad libitum* for 5 days. On the 5th day, mice were placed in DD for 24 h and then euthanized.	Gene expression by qPCR
Oishi et al., 2009a [26]	Male C57Bl/6J and *Per2* transgenic (overexpressing rat PER2) mice.	To investigate the function of PER2 in the circadian regulation of fibrinolysis and obesity-induced hypofibrinolysis.	Normal diet (CE-2; Clean Japan Inc., Tokyo, Japan); high-fat/high-sucrose diet (with 54.5% fat, 28.3% carbohydrates, and 17.2% protein; F2HFHSD; Oriental Yeast Co., Ltd., Tokyo, Japan)	Mice were fed with normal diet, or HFSD for 12 weeks to induce obesity and then euthanized.	Gene and protein expression by qPCR and Western blotting, respectively; measurement of blood metabolic parameters; measurement of plasma and tissue PAI-1; stimulation with 10 ng/mL TGF-β1.
Oishi et al., 2009b [32]	Male Jcl:ICR mice.	To investigate the effect of a ketogenic diet on fibrinolytic functions.	Normal diet (CE-2; 12.6% fat, 58.3% carbohydrate, and 29.3% protein, Clea Japan Inc); ketogenic diet (KD; 94.8% fat, 0.1% carbohydrate, and 4.8% protein, w/w; modified AIN-93G; Oriental Yeast Co. Ltd.).	Mice were fed with normal or KD under LD 12:12 for 14 days. Mice were euthanized and the tissues were collected. Mice with control or KD stayed under LD 12:12, and then they were transferred to constant DD.	Gene expression by qPCR; measurement of blood metabolic parameters and plasma PAI-1; drinking behavior analyses by Chronobiology Kits (Stanford Software Systems).
Hou et al., 2009 [24]	Male *ApoE*^−/−^, C57Bl/6J mice.	To investigate the effect of hyperlipidemia on the expression of circadian genes in an atherosclerotic mouse model.	Regular chow diet, high-fat diet (HFD with 0.15% cholesterol and 21% fat).	*ApoE*^−/−^ mice were fed with regular diet or HFD, and C57Bl/6J mice were fed with regular diet in an LD cycle for 2 weeks. Mice were transferred to a DD cycle for 3 weeks and then euthanized.	Analysis of serum lipid, analyses of atherosclerotic plaques or foam cells by oil red O staining, gene expression by qPCR.
Tsai et al., 2010 [27]	Male WT, CCM mice	To investigate the role of the cardiomyocyte circadian clock in the metabolic adaptation of the heart to chronic high-fat feeding.	Standard murine diet (Teklad Lab Animal Diets, Harlan Laboratories, Indianapolis, IN), HFD (with 45% fat, Research Diets, New Brunswick, NJ), control diet (with 10% fat, Research Diets, New Brunswick, NJ)	Mice were fed with a standard diet, HFD, or control diet for 16 weeks *ad libitum* and then euthanized.	Echocardiography; plasma hormone, substrate and AMPK activity measurements; microarray analyses; gene and protein expression by qPCR and Western blotting, respectively; myocardial triglyceride content measurements; isolated working mouse heart perfusions; lipid extraction, fractionation, and profiling; analyses of heart sections by oil red O staining.
Oishi et al., 2010 [25]	Male *Ppar*α-null, WT mice.	To investigate the role of PPARα in ketogenic diet-induced PAI-1 gene expression.	Normal diet (AIN-93M, Oriental Yeast Co., Ltd., Tokyo, Japan), ketogenic diet (KD; 94.8% fat, 0.1% carbohydrate and 4.8% protein, w/w; modified AIN-93G; Oriental Yeast Co Ltd.).	Mice were fed with normal diet supplemented with bezafibrate or a KD. The PPARγ antagonist bisphenol A diglycidyl ether was added to diets for 5 days. Mice were euthanized and the tissues were collected.	Gene expression by qPCR; measurement of plasma PAI-1.
Bray et al., 2013 [20]	Male C57Bl/6J mice.	To investigate the effects of RF during the light (sleep) phase on body weight gain, diurnal variation in energy balance, gene expression, and humoral factors.	Standard rodent diet (Harlan NIH-31 irradiated open formula mouse/rat diet, 4.7% fat)	Mice were fed with a standard diet under light-phase (ZT0-ZT12) or dark-phase (ZT12-ZT24) RF during 9 days and then euthanized.	Continuous 24-h monitoring of behavioral and physiological parameters; gene expression by qPCR; humoral factor measurements
Wang et al., 2015 [28]	Female C57Bl/6J mice, and 35 male pups postnatal day 17.	To investigate the effect of maternal obesity on the circadian clock and metabolism in the heart and liver of the offspring.	Normal diet (ND, D12450B, fat content 10%; Research Diets Inc., New Brunswick, NJ, USA) or HFD (D12492, fat content 60%)	The animals were fed normal diet or HFD for 6 weeks until mating, during gestation and lactation (until day 16 of lactation). On day 16 of lactation, both groups were fed ND. The pups were suckled until day 21 of life and then fed ND until the day of euthanasia.	Gene and protein expression by qPCR and Western blot, respectively; measurement of the body weight; serological analyses.
Noyan et al., 2015 [22]	Male C57Bl/6J mice.	To investigate the effect of the short-term, mild CR before induction of experimental MI to protect the heart from ischemic injury and to understand the underlying molecular pathways.	Regular chow diet or CR (30% less than the calculated mean daily AL food consumption)	Mice were fed *ad libitum*, stressed AL diet, or CR diet for 7 days, prior to MI via permanent coronary ligation and then euthanized.	Protein and gene expression by Western blotting; mRNA and microRNA analyses; signaling assessments in myocardial tissue; pathway enrichment analysis; infarct size (2 days post-MI); cardiac hemodynamics (before and 2 days post-MI); protein abundance of caspase.
Mukherji et al., 2015 [21]	Male C57Bl/6J, *Pparα*^hep−/−^, *Ppar*α^iec−/−^, *Bmal1*^hep−/−^, *Bmal1*^iec−/−^, *RevErb*α^hep−/−^, *RevErbα*^iec−/−^, adrenalectomized mice	To investigate molecular mechanisms generated by shifting feeding to the rest phase and how this environmental cue alters the expression of circadian clock components, thereby leading to obesity and metabolic syndrome-like pathology.	Control diet	Mice were fed with a control diet *ad libitum* or RF during the 12 h light phase (ZT0-ZT12) under LD 12h:12h.	Isolation of IECs; determination of RNA transcripts; protein immunoblots; measurement of NAD^+^; FGF21 measurement; chip assays; plasma metabolic analysis.
Oishi et al., 2017 [29]	Male C57Bl/6J mice	To investigate the involvement of feeding cycle-dependent endogenous insulin rhythms in the circadian regulation of peripheral clocks, and the effect of exogenous insulin on the expression of clock genes.	High-fat/high-sucrose diet (F2HFHSD; Oriental Yeast Co., Ltd., Tokyo, Japan), normal diet (Clea Japan Inc., Tokyo, Japan).	Mice were fed HFSD for 8 h during nighttime (ZT14-22) or daytime (ZT2-10) for one week. After this period mice were euthanized and the tissues were collected.	Measurement of blood hormones; gene expression by qPCR; assay of phosphorylated AKT; wheel-running activity analyses.
Marques et al., 2017 [33]	Male C57Bl/6J mice	To investigate the effect of a high-fiber diet and supplementation with the short-chain fatty acid (acetate) on the gut microbiota and the prevention of cardiovascular disease.	Control diet (normal chow, 47.6%), high-fiber diet (72.7% fiber, SF11-025; Specialty Feeds, Perth, Australia), or SCFA supplementation (200 mmol/L magnesium acetate, Meck Millipore, 1.05819.1000)	Mice were fed with a high-fiber diet or acetate supplementation for 3 weeks before sham or DOCA surgery.	Morphological analyses in the heart, kidney, and lung; histological analyses in the heart and kidney; bioinformatic analyses; renal and cardiac transcriptome; analyses of the composition of the gut microbiota.
Noguchi et al., 2018 [34]	Male C57Bl/6J, *Per2*:Luciferase knock-in, A/J, C3H/HeJ mice.	To investigate whether the levels of systemic phosphate regulate the circadian cycle in peripheral tissues.	Normal diet (with 0.4% phosphate), low phosphate diet (with 0.06% phosphate; Teklad 2018; 0.65% phosphorus).	Mice were fed with normal diet or low phosphate diet. The phosphate-restricted diet group was fed two days before fracture and for 16 more days. A normal diet was reintroduced afterward.	Circadian rhythm studies were performed 10 days post-fracture; serum analyses; analyses of cartilage and bone by CECT and µCT; gene expression by qPCR; microarray analyses; analysis of periodicity of *Per2* in callus organ culture.
Reitz et al., 2020 [30]	Male *Clock*^Δ19^^/Δ19^ and WT mice.	To investigate whether *Clock*^Δ19/Δ19^ mice develop or are resilient to obesity induced cardiovascular disease.	HFD (45% fat, 20% protein, 35% carbohydrate, TD.06415, Envigo Teklad Diets), normal standard chow diet (10% fat, 20% protein, 70% carbohydrates, TD.08806, Envigo Teklad Diets)	Mice were fed with HFD or normal standard chow diet for 24 weeks and then euthanized.	CLAMS for evaluation of food/calorie intake and metabolic parameters; metabolic measurements; morphometric, hemodynamics, histological analyses; echocardiography; gene and protein expression by qPCR and Western blotting, respectively; microarray and bioinformatics analyses.
Mia et al., 2021 [31]	Male C57Bl/6J mice	To investigate diurnal metabolic inflexibility in the heart in obesity.	Control diet (10% calories from fat, Research Diets, New Brunswick, NJ; catalog number D12450K) or HFD (45% calories from fat, Research Diets, New Brunswick, NJ; catalog number D12451)	Mice were fed with control or HFD *ad libitum* for 18 weeks, followed by access only during the 12 h dark phase for 2 weeks.	Behavioral and metabolic monitoring; histological analysis, transcriptome, echocardiographic image, gene and protein expression, lipidomic analyses; NEFA quantification; isolated heart perfusion.
Xin et al., 2021 [23]	Male and female C57Bl/6J mice	To investigate the transition kinetics during inverted feeding.	Normal chow diet (Rodent maintenance diet; Hunan SJA Laboratory Animal Co. LTD).	Female mice were fed with normal chow diet for 7 days, following DRF or NRF for 7 or 36 days. They were submitted to LL for 9 days, following DRF and LL for 9 additional days. Male mice were fed with normal chow diet for 7 days, following DRF or NRF for 7 additional days. After this period, female and male mice were euthanized.	Transcriptome and metabolomic profiling; food intake, body weight, and locomotor activity analyses; global profiling of transcripts; untargeted metabolomics; targeted lipidomics; Acyl-CoA quantification by LC/MS; gene expression by qPCR; transcriptome analyses; circadian rhythmicity analyses; phase set and cistrome enrichment analyses; heatmap of expression profile.
Murata et al., 2021 [35]	*BMAL1-BioID*, C57Bl/6J, homozygous 540 EGFP-pA^flox^ mice	To investigate the effect of biotin diet on protein biotinylation in several tissues in the *BMAL1-BioID* mouse model.	Chow diet, high-biotin diet (0.5% biotin; Fujifilm Wako Pure Chemical, #021-08712).	Mice were fed with a biotin-rich diet or chow diet *ad libitum* for 7 days and then euthanized.	Protein expression by Western blotting; histological analyses; streptavidin blot analysis; biotin labeling assay *in vitro;* co-immunoprecipitation.
Latimer et al., 2021 [36]	Male C57Bl/6J, CBK, CON mice	To investigate the effect of time of day of dietary BCAA consumption on physiological responses (cardiac growth) and its pathological implications	Low BCAA diet (Teklad TD.150662 custom diet; with leucine, isoleucine, and valine 3-fold lower than the standard diet), high BCAA diet (Teklad TD.170323 custom diet; with leucine, isoleucine, and valine 2-fold higher than the standard diet) or a standard diet (Teklad TD.170323 custom diet).	The dietary intervention occurred acutely and chronically. Acute intervention: mice were fed with an early high BCAA diet or early low BCAA diet, and a late high BCAA diet or late low BCAA diet for 4 h. Chronic intervention: mice were fed with an early high BCAA diet, and a late high BCAA diet for 4 weeks or 6 weeks.	Transverse aortic constriction; CLAMS for evaluation of food/calorie intake, energy expenditure, and physical activity; spectrometry (plasma BCAA levels); quantitative magnetic resonance imaging (lean and fat body mass); gene and protein expression by qPCR and Western blot, respectively; histological evaluation.

Legend: PPARα, alpha isoform of peroxisome proliferators-activated receptors; mRNAs, messenger ribonucleic acids; qPCR, quantitative polymerase chain reaction; *Dbh*^+/−^, mice expressing dopamine hydroxylase; *Dbh*^−/−,^, dopamine hydroxylase knockout mice; SCN, suprachiasmatic nucleus; RF, restricted feeding, LD, light-dark cycle; DD, constant darkness; *Per2*, period circadian regulator 2; F2HFHSD, high-fat high-sucrose diet; PAI- 1, plasminogen activator inhibitor-1; TGF- β2, transforming growth factor-β1; KD, ketogenic diet; *ApoE^−/−^* mice, apolipoprotein E-deficient mice; HFD, high-fat diet; CCM mice, cardiomyocyte clock mutant mice; ZT, zeitgeber time; AMPK, adenosine monophosphate activated protein kinase; *Ppar*α-*null* mice, homozygous *Pparα*-null mice; PPARα, peroxisome proliferator-activated receptor α; PPARγ, peroxisome proliferator-activated receptor γ, NIH-31, Irradiated Open Formula Mouse/Rat Diet 4.7% fat; ND, normal diet; CR, caloric restriction; AL, *ad libitum*; microRNA, micro ribonucleic acid; MI, myocardial infarction; *Pparα*^hep−/−^ mice, mice with selective *Ppar*α mutations in liver; *Ppar*α^iec−/−^ mice, mice with selective *Ppar*α mutations in intestinal epithelial cells; *Bmal1*^hep−/−^ mice, mice with selective *Bmal1* mutations in liver; *Bmal1*^iec−/−^ mice, mice with selective *Bmal1* mutations in intestinal epithelial cells; *Rev-Erb*α^hep−/−^ mice, mice with selective *Rev*-*Erbα* mutations in liver; *Rev-Erbα*^iec−/−^ mice, mice with selective *Rev*-*Erbα* mutations in intestinal epithelial cells; IECs, intestinal epithelial cells; NAD^+^, nicotinamide adenine dinucleotide oxidized; FGF21, fibroblast growth factor 21; AKT, protein kinase B; SCFA, short-chain fatty acid; DOCA, deoxycorticosterone acetate; CECT, contrast-enhanced computed tomography; µCT, µ computed tomography; *Clock*^Δ19/Δ19^ mice, mice with deletion of exon 19 in the *Clock* gene; CLAMS, comprehensive laboratory animal monitoring system; NEFA, non-esterified fatty acid; DRF, daytime-restricted feeding; NRF, nighttime-restricted feeding; LL, constant light; LC/MS, chromatography in combination with tandem mass spectrometric detection; *Bmal1-BioID*, brain and muscle ARNT-like 1 (BMAL1)-BirA* knock-in mice; BMAL1, basic helix-loop-helix ARNT like 1; CBK, cardiomyocyte-specific *Bmal1* knockout; CON mice, littermate control mice; BCAA, branched-chain amino acids.

**Table 3:** Authors, year of publication, and the results obtained.

Dietary Interventions/Diet	Author/Year	Alterations in the Circadian Clock/Gene Expression of the Heart	Alterations in the Circadian Clock/Gene Expression of the Other Tissues	Positive (+), Negative (−) or Neutral (N) Effect of the Diet/Dietary Intervention
Restricting feeding	Goh et al., 2007 [18]	WT mice with RF: shifted the acrophase of circadian gene expression by 5.8 ± 2.0 h and reduced the amplitude of *Bmal1*. *Ppar*α-null mice with RF: shifted the acrophase of circadian gene expression 14.2 ± 2.5 h, reduced the amplitude *Rev*-*erbα,* and increased that of *Bmal1*.	WT and *Pparα*-null mice: similar expression profiles and amplitudes for *Per1* and *Per3* genes with mean acrophase of ZT11.4 and ZT10.6, respectively (liver, BAT, and eWAT) reflecting a 10 h time difference from *Bmal1* and *Npas2*.	(−)
Restricting feeding	Reilly et al., 2008 [19]	*Dbh^−/−^* mice with RF: no changes in the expression rhythms of *Per1/2*, *Dbp* and *E4bp4* genes. Expression levels of clock genes were similar to those of *Dbh^+/−^* mice. Treatment with α and β adrenergic receptor antagonists: no changes in the rhythmicity of *Per1*, *Dbp*, *E4bp4,* and *Bmal1* genes in both genotypes.	*Dbh^−/−^* mice with RF: no changes in the expression rhythms of *Per1*, *Per2*, *Dbp* and *E4bp4* genes (aorta and liver). Treatment with α and β adrenergic receptor antagonists: no changes in the rhythmicity of *Per1*, *Dbp*, *E4bp4,* and *Bmal1* genes (aorta, liver, and BAT) in both genotypes.	(N)
High-fat, high-sucrose diet	Oishi et al., 2009a [26]	*Per2*/Tg mice fed ND: oscillation in *Per2* mRNA expression levels in the light phase. *Per2*/Tg mice with HFSD: overexpression of *Per2* mRNA.	*Per2*/Tg mice with ND or HFSD: overexpression of *Per2* mRNA and attenuation of *Bmal1* mRNA expression (liver and adipose tissue).	(N)
Ketogenic diet	Oishi et al., 2009b [32]	Jcl:ICR mice with KD: advancing acrophase expression of *Per2* and *Dbp* genes.	Jcl:ICR mice with KD: advancing acrophase expression of *Per2* and *Dbp* genes (liver, kidney, and adipose tissues).	(−)
High-fat diet	Hou et al., 2009 [24]	WT mice fed regular diet: higher *Bmal1* and *Per2* mRNA expression levels in CT20 and CT8, respectively. Lower levels in CT8 and CT20, respectively. *ApoE*^−/−^ mice fed HFD: higher levels of *Bmal1* and *Per2* mRNA in CT0 and CT12, respectively. Lower levels in CT12 and CT0, respectively. No changes in *Cry1* mRNA expression levels in both genotypes on regular diet.	WT mice fed regular diet: higher levels of *Bmal1*, *Cry1,* and *Per2* mRNA expression in CT20, CT8, and CT12, respectively. *ApoE*^−/−^ mice fed HFD: higher levels of *Bmal1*, *Per2,* and *Cry1* mRNA expression in CT12.	(−)
High-fat diet	Tsai et al., 2010 [27]	†	†	†
Ketogenic diet	Oishi et al., 2010 [25]	*Pparα*-null and WT mice with KD: phase-advanced circadian expression for *Bmal1* and low average levels of *Dbp* expression.	The expression of *Bmal1* was phase-advanced in the liver.	(−)
Restricted feeding	Bray et al., 2013 [20]	WT mice with RF: altered expression of clock genes (*Bmal1*, *Per2*, *Cry2,* and *Rev*-*Erbα*) and clock-controlled genes (*Dbp*); lower and less consistent effects on the phases of clock component and output gene oscillations; average phase shifts and repression of amplitude were 3.90 ± 0.83 h and 54 ± 5%, respectively.	WT mice with RF: changes in expression of clock genes (*Bmal1*, *Per2*, *Cry2,* and *Rev*-*erbα*) and clock-controlled genes (*Dbp*); dramatic phase shifts in gene expression of clock components and output genes (liver); phase differences within the range of 6 and 11 h (mean 8.38 ± 0.84 h) (liver); smaller and less consistent effects on phase shifts of clock and output gene components (epididymal fat and gastrocnemius muscle); average phase shifts and repression of amplitude were 6.88 ± 2.06 h and 69 ± 3% (epididymal), 3.46 ± 1.41 h and 24 ± 20% (gastrocnemius muscle), respectively.	(−)
High-fat diet	Wang et al., 2015 [28]	P17 pups from HFD-fed dams: higher mRNA levels of *Bmal1* in ZT1 with a circadian pattern and oscillates in antiphase for the *Per2* gene. P35 pups from HFD-fed dams: improved phase changes while maintaining amplitude defects.	P17 pups from HFD-fed dams: *Fas* exhibited a rhythmic expression pattern in control animals, which peaked at ZT9, the late stage of the light phase. *Pgc*-1α exhibited a significantly rhythmic and lower expression (liver). P35 pups from HFD-fed dams: abolished the circadian expression rhythm of *Fas* and *Pgc-1α* (liver).	(−)
Restricted feeding	Noyan et al., 2015 [22]	WT mice under short-term CR: modulation in the mRNA profiles of pre-MI genes associated with the circadian clock.	WT mice under short-term CR: global change in gene expression associated with oxidative stress, immune function, apoptosis, metabolism angiogenesis, cytoskeleton and extracellular matrix.	(+)
Restricted feeding	Mukherji et al., 2015 [21]	WT mice with RF 8 (day 8): altered the expression of *Rev-Erbα*, *Per1, and Per2* genes.	WT mice with RF1 (day 1): increased expression of *Per1*, *Per2,* and *Rev*-*Erbα* genes; no changes in expression of *Bmal1*, *Cry1,* and *E4bp4* (liver, IECs, and pancreas). RF2 (day 2): changes in expression of clock components (*Per1*, *Per2*, *Rev*-*Erbα*, *Bmal1*, *Cry1* e *E4bp4*); no recruitment of Bmal1 to the E-box region as a result of Rev-Erbα repression. After 4, 8, 15, 30, and 90 days of RF: change in expression levels of *Per2*, *Per3*, and *Rev*-*Erbα* genes (liver and intestinal epithelial cells). Glucose administration did not affect the expression pattern of *Per1*, *Per2*, and *Rev*-*Erbα* genes (liver, intestine, and pancreas).	(−)
High-fat, high-sucrose diet	Oishi et al., 2017 [29]	WT mice with RF: no changes in clock gene expression in both groups. Exogenous insulin administration: significantly increased the expression of the clock genes, *Per1* and *Per2*.	WT mice with RF in NF: phase advancement of *Per2* and Dbp genes (liver). Exogenous insulin administration: increased expression levels of *Per1* and *Per2* genes (liver, lung, WAT, and skeletal muscle).	(N)
High-fiber diet	Marques et al., 2017 [33]	WT mice fed high-fiber diet: upregulation of circadian rhythm in the cardiac transcriptome (q = 0.021). WT mice supplemented with acetate: upregulation of circadian rhythm in the cardiac transcriptome (q = 0.086).	WT mice fed high-fiber diet: upregulation of circadian rhythm in the renal transcriptome. WT mice supplemented with acetate: upregulation of circadian rhythm in the renal transcriptome.	(+)
Low phosphate diet	Noguchi et al., 2018 [34]	Mice with hypophosphatemia diet: higher expression levels of *Per1*, *Per2*, *Per3,* and *Bmal1* genes in ZT9, ZT9, Zt16-5, and ZT21, respectively. Mice with control diet: higher expression levels of *Per1*, *Per2*, *Per3*, *Bmal1,* and *Cry1* genes in ZT12, ZT15, ZT9-18, ZT24, and ZT15-24, respectively.	Mice fed hypophosphatemic diet: higher expression levels of *Per2*, *Bmal1,* and *Cry* genes in ZT6-12, ZT6-9, ZT18-ZT24, and ZT15-21 (callus and growth plate tissues) Mice fed control diet: higher expression levels of *Per1*, *Per2, Per3, Bmal1,* and *Cry* genes in ZT12, ZT15, ZT15, ZT21, and ZT15-21 (callus and growth plate tissues)	(−)
High-fat diet	Reitz et al., 2020 [30]	The circadian mechanism is involved in the transcriptional responses of oxidative stress and antioxidant pathways.	†	(N)
High-fat diet	Mia et al., 2021 [31]	WT mice with HFD: no changes in circadian clock components (*Bmal1*, *Clock*, *Npas2*, *Nr1d1*, *Nr1d2*, *Per2*, *Per3*, and *Cry2*).	†	(N)
Restricting feeding	Xin et al., 2021 [23]	Female mice on RF for 36 days: phase-locked to LD cycles. Female mice on LL cycle for 9 days: reversed the phase of clock genes. Cardiac transcriptome showed resistance in phase drag by reversed feeding and the fatty acid rhythm was entrained.	Female mice on RF for 36 days: phase shift of clock genes similar to animals on 7 days RF (liver and adipose tissue). Female mice on LL cycle for 9 days: increased the behavioral rhythm by 1.7 h, and did not change the phase of clock genes (liver). Induced oscillations in clock genes (adipose tissue); reversed the phase of clock genes (kidney). The liver and adipose tissue transcriptomes were entrained by the reversed feeding. The kidney transcriptome is more resistant to phase drag by reversed feeding.	(−)
High-biotin diet	Murata et al., 2021 [35]	BMAL1-BioID mice with biotin-rich diet: reduced endogenous BMAL1 expression.	BMAL1-BioID mice on biotin-rich diet: reduced endogenous BMAL1 expression. BMAL1-BioID mice on biotin-rich diet: reduced endogenous BMAL1 expression (liver and kidney) and showed endogenous CLOCK biotinylation in a diet-dependent manner (liver and brain).	(N)
Low BCAA diet	Latimer et al., 2021 [36]	CBK mice: time-of-day fluctuations in mRNA levels of *Arntl*, *Nr1d1*, and *Dbp* were attenuated (59%–69% reduction in amplitude).	†	(N)

Legend: WT, wild-type mice; *Pparα*-null mice, homozygous *Pparα*-null mice; *Bmal1*, brain and muscle ARNT-like 1; *Per3*, period 3; *Rev-erbα*, nuclear receptor subfamily 1, group D, member 1; *Npas2*, neuronal PAS domain protein 2; BAT, brown adipose tissue; WAT, white adipose tissue; *Per1*, period 1; ZT, zeitgeber time, hours after lights are turned on; *Dbh^−/−^*, dopamine-hydroxylase knockout mice; *Per2*, period 2; *Dbp*, D site binding protein; *E4bp4,* interleukin 3 regulated; *Dbh^+/−^*, mice expressing dopamine hydroxylase; *Clock*, circadian locomotor output cycles kaput; ASMCs, aortic smooth muscle cells; β2 AR, β2 antagonist receptor; α1 AR, α1 agonist receptor; mRNA, messenger ribonucleic acids; *Per2/Tg* mice, transgenic mouse model overexpressing *Per2* mice; ND, normal diet; *Bmal2*, brain and muscle ARNT-like 2; PAI-1, plasminogen activator inhibitor-1; CT, clock time; *ApoE^−/−^* mice, apolipoprotein E-deficient mice; *Cry1*, cryptochrome 1; SCN, suprachiasmatic nucleus; HFD, high-fat diet; RF, restricted feeding; *Cry2*, cryptochrome 2; P17, 17 postnatal days; P35, 35 postnatal days; *Fas*, fatty acid synthase; *Pgc-1α*, peroxisome proliferator-activated receptor gamma coactivator 1-alpha; pre-MI, pre-myocardial infarct; RF, restricted feeding; IECs, intestinal epithelial cells; E-box, enhancer box; NF, nighttime; *Nr1d1*, nuclear receptor family 1, member D1; *Nr1d2*, nuclear receptor family 1, member D2; LD, Light/dark cycles LL, constant light; *BMAL1-BioID*, brain and muscle ARNT-Like 1 (BMAL1)-BirA* knock-in mice; CBK, cardiomyocyte-specific *Bmal1* knockout; *Arntl*, Aryl hydrocarbon receptor nuclear translocator-like protein 1. † Did not present results.

**Table 4:** Authors, year of publication, and the results obtained.

Diet Interventions/Diet	Author/Year	Metabolic Changes	Positive (+), Negative (−), or Neutral (N) Effect of the Diet/Dietary Intervention
Restricting feeding	Goh et al., 2007 [18]	RF reduced food intake. In WT mice under conditions *ad libitum*, the *Pparα* mRNA levels showed a statistically significant circadian profile.	(+)
Restricting feeding	Reilly et al., 2008 [19]	†	†
High-fat, high-sucrose diet	Oishi et al., 2009a [26]	WT and *Per2*/Tg mice with HFSD: increased body weight, plasma insulin, total cholesterol, and insulin/glucose ratio. PAI-1 mRNA expression levels did not differ. *Per2*/Tg with HFSD or ND mice: dampening of PAI-1 expression rhythms in the heart.	(−)
Ketogenic diet	Oishi et al., 2009b [32]	Jcl:ICR mice with KD: reduced body weight and glucose levels, increased plasma levels of FFA and total ketone bodies, increased PAI-1 mRNA levels (heart and liver) with advanced acrophase to 4.7 h (heart), 7.9 h (kidney), and 7.8 h (epididymal fat), and led to phase advancement of the endogenous circadian clock that governs rhythmic behavior.	(-)
High-fat diet	Hou et al., 2009 [24]	*ApoE*^−/−^ mice fed HFD: increased total and LDL-c cholesterol levels, decreased HDL-c levels, with peak concentrations in CT12, led to atherosclerotic plaque formation in the aortic root, increased diurnal expression levels of lipid metabolism-related transcription factors *Pparα* and *Rxrα*.	(−)
High-fat diet	Tsai et al., 2010 [27]	CCM mice fed HFD: increased caloric intake; body weight gain with increased body fat percentage; altered metabolism gene expression, cardiac triglyceride and lipid species. CCM mice fed standard diet: attenuation in gene and protein expression of myocardial enzymes related to triglyceride metabolism. HFD at the end of the active phase: increased levels of triglyceride, lipid and fatty acid metabolism genes (*Dgat2*, *Agpat3*, *Hsl*, S3-*12*, *Ucp3* and *Pdk4*), and in the rate of substrate utilization.	(−)
Ketogenic diet	Oishi et al., 2010 [25]	*Pparα*-null mice fed KD: decreased BW, plasma FFA levels, and total ketone bodies; induced fatty liver; and increased hepatic total cholesterol levels. WT mice fed KD: increased circadian expression of PAI-1 mRNA (heart and liver). Benzafibrate: induced the expression of the *Pai-1*, *Cy4A10*, and *Fgf21* genes in a PPARα-dependent manner.	(−)
Restricted feeding	Bray et al., 2013 [20]	WT mice on RF: changes in whole-body energy balance, higher food intake during DP, little influence on physical activity rhythms, diurnal variation in plasma glucose and triglyceride levels, body weight gain, and tissue-specific changes in metabolic genes (*Accα*, *Glut2*, *Lpk*, *Lgs*, *Mcad*, *Dgat2*, *Acsl1*, *Atgl*, *Lipe*, *Mcp1*, *Glut4*, *Pdk4*).	(−)
High-fat diet	Wang et al., 2015 [28]	Pregnant female mice fed HFD: hypercholesterolemia and hyperlipidemia. Pups of obese dams: expressed inflammatory cytokines (*Tnfα* and *Il-6*), showed an anti-phase pattern for *Pparα* and *Cpt1b* genes.	(−)
Restricted feeding	Noyan et al., 2015 [22]	WT mice under short-term CR: smaller infarct size and apoptosis, improved cardiac function at 2 d post-MI and survival and weight loss, and modulated signaling pathways associated with cardio-protection, mitochondrial function, and biogenesis.	(+)
Restricted feeding	Mukherji et al., 2015 [21]	WT mice with RF: increased ketone bodies, FFA, glucagon and corticosterone; decreased insulin and glucose levels stimulated the expression of *Pparα*, *Creb,* and *Rev-Erbα*, increased GSK3β activity. Glucose administration prevented plasma glucose reduction; increased levels of FFA, ketone bodies, corticosterone, FGF21; and inhibited transcription of *Pparα* and *Rev-Erbα*.	(−)
High-fat, high-sucrose diet	Oishi et al., 2017 [29]	WT mice with DRF: increased plasma insulin and GLP-1 concentration; did not affect plasma corticosterone and GIP levels. Exogenous insulin administration: induced Akt phosphorylation (heart, liver, and skeletal muscle).	(+)
High-fiber diet	Marques et al., 2017 [33]	WT mice with high-fiber diet: altered the composition of the gut microbiota and increased acetate levels. WT mice supplemented with acetate: altered the composition of the intestinal microbiota and increased the percentage of acetate-producing bacteria. WT mice with high-fiber diet and acetate: reduced systolic and diastolic BP levels, altered the renal transcriptome (*Rasal1*, *Cyp414,* and *Cck*) of genes related to renal fibrosis, fluid absorption through sodium channel regulation, anti-inflammatory action, and the cardiac transcriptome (*Tcap* and *Timp4*) of genes related to cardiac diseases, pathways acting on cell cycle, replication, translation, mRNA metabolism, respiratory electron chain, mitogen-activated protein kinase signaling, and renin-angiotensin system.	(+)
Low-phosphate diet	Noguchi et al., 2018 [34]	Mice with hypophosphatemic diet: increased total cartilage volume fraction, reduced TMD and BV/TV, increased osteochondromic progenitor lineage and impaired chondrocytes, reduced the size of proliferative matrix-forming cells, and affected systemic regulation of mineral metabolism. Transcriptome analyses revealed that 1.879 genes associated with diet and having a circadian pattern of regulation, those with mitochondrial function, including oxidative metabolism and canonical regulatory pathways associated with apoptotic signaling.	(−)
High-fat diet	Reitz et al., 2020 [30]	*Clock*^Δ19/19^ mice fed HFD or SC: hypercholesterolemia, hyperglycemia, hyperinsulinemia, increased body weight and epididymal white adipose tissue, no changes in cardiomyocyte hypertrophy, normal cardiac function, and structure. No changes in the expression of genes related to cardiac remodeling and oxidative stress (*Nampt*, *Sirt1*, *Sirt2*, *Sirt3*, *Sirt4*, *Sirt6*, *Foxo1*, and *Foxo3*). Increased concentrations of CAT and GPx proteins, and *Pparγ* gene transcripts.	(N)
High-fat diet	Mia et al., 2020 [31]	HFD increased body weight, adiposity, daily energy expenditure, reduced physical activity, and RER, increased stroke volume, left posterior ventricular wall thickness during systole, the BVW/TL ratio, cardiomyocyte area, cardiac fibrosis, and altered the temporal regulation of the cardiac transcriptome, especially of metabolism-related genes. Day–night differences affected cardiac glucose oxidation, lactate release, and the cardiac lipidome. The diurnal rhythms of lipid metabolism genes (*Cd36*, *Mcd*, *Lcad*, and *Lipe*) and plasma levels were altered. Time-of-day-restricted feeding restored body metabolic rhythms, normalized the adverse effects of cardiac remodeling (BVW/TL ratio, cardiomyocyte size, cardiac fibrosis, cardiac steatosis), and increased the day–night difference in cardiac lipid metabolism.	(−)
Restricting feeding	Xin et al., 2021 [23]	There was no change in body and adipose tissue weight after 1 week of RF.	(N)
High-biotin diet	Murata et al., 2021 [35]	A 0.5% biotin diet caused protein biotinylation in the brain, heart, testis, and liver of *Bmal1-BioID* mice.	(+)
Low BCAA diet	Latimer et al., 2021 [36]	Mice fed low BCAA diet: decreased body mass, adiposity, and modest cardiac atrophy. Consumption of dietary BCAAs at the end of the active period (dark): increased the cardiomyocyte size, promoted cardiac protein synthesis, and highly dynamic cardiac growth.	(+)

Legend: RF, restricted feeding; WT, wild-type mice; *Pparα*, peroxisome proliferators-activated receptors; mRNA, messenger ribonucleic acids; HFSD, high-fat/high-sucrose diet; *Per2*/Tg, transgenic mouse model overexpressing *Per2* mice; PAI-1, plasminogen activator inhibitor-1; ND, normal diet; KD, ketogenic diet; FFA, free fatty acid; LDL-c, low-density lipoprotein cholesterol; HDL-c, high-density lipoprotein cholesterol; CT, circadian time; *Rxrα*, retinoid X alpha receptor; HFD, high-fat diet; *ApoE*^−/−^, apolipoprotein E-deficient mice; CCM, cardiomyocyte clock mutant; *Dgat2*, diacylglycerol O-acyltransferase 2; *Hsl*, lipolysis; *Agpat3*, 1-acylglycerol-3-phosphate O-acyl- transferase 3; *S3-12*, lipid droplet-binding protein; *Ucp3*, mitochondrial uncoupling protein 3; *Pdk4*, pyruvate dehydrogenase lipoamide kinase isoenzyme 4; BW, body weight; *Fgf21*, fibroblast growth factor 21; DP, dark-phase; *Accα*, acetyl-CoA carboxylase alpha; *Glut2*, facilitated glucose transporter 2; *Lpk*, liver pyruvate kinase; *Lgs*, liver glycogen synthase; *Mcad*, medium chain acyl-CoA dehydrogenase; *Acsl1*, acyl-CoA synthetase long-chain family member 1; *Atgl*, adipose triglyceride lipase; *Lipe*, hormone sensitive lipase; *Mcp1*, carnitine palmitoyltransferase 1B; *Glut4*, glucose transporter 4; *Tnfα*, tumor necrosis factor alpha; *Il*-6, interleukin-6; *Cpt1b*, carnitine palmitoyltransferase 1b; CR, caloric restriction; MI, myocardial infarction; CREB, cAMP response element-binding protein; FGF21, fibroblast growth factor 21; GSK3β, glycogen synthase kinase 3β; *Rev*-*Erbα*, nuclear receptor subfamily 1, group D, member 1; *Bmal1*, brain and muscle ARNT-Like 1; GLP-1, glucagon-like peptide-1; DRF, daytime-restricted feeding; GIP, glucose-dependent insulinotropic polypeptide; Akt, protein kinase B; BP, blood pressure; *Rasal1*, RAS protein activator like 1; *Cyp4a14*, Cytochrome P450 omega-hydroxylase 4a14; *Cck*, cholecystokinin; *Tcap*, Titin-cap; *Timp4,* Tissue metallopeptidase inhibitor 4; TMD, total mineral density; BV/TV, bone volume ratio; SC, normal standard chow diet; *Clock*^Δ19/19^, mice with deletion of exon 19 in the *Clock* gene; *Nampt*, nicotinamide phosphoribosyl transferase; *Sirt1*, sirtuin 1; *Sirt2*, sirtuin 2; *Sirt3*, sirtuin 3; *Sirt4*, sirtuin 4; *Sirt6*, sirtuin 6; *Foxo1,* fork head 1; *Foxo3*, fork head 3; CAT, cardiac catalase; GPx, glutathione peroxidase; *Pparγ*, peroxisome proliferator-activated receptors; RER, respiratory exchange ratio; BVW/TL, biventricular weight to tibia length ratio; *Cd36*, fatty acid translocase; *Mcd*, malonyl-CoA decarboxylase; *Lcad*, long chain acyl-CoA dehydrogenase; *Lipe*, hormone-sensitive lipase; *Bmal1-BioID*, brain and muscle ARNT-Like 1 (*Bmal*1)-BirA* knock-in mice; BCAA, branched-chain amino acids. † Did not present result.

## Supplementary Materials

The following supporting information can be downloaded at: https://www.mdpi.com/article/10.3390/metabo12121273/s1, Table S1: diets and their effects on the circadian clock; Figure S1: result of the assessment of the risk of bias of the studies included in the systematic review. (a) Summary of the risk of bias; (b) risk of bias graph.
