# Supplementary material for: The Effect of Diet on the Cardiac Circadian Clock in Mice: A Systematic Review
Source: Metabolites. 2022 Dec 15;12(12):1273. doi: 10.3390/metabo12121273 (PMC9786298; doi:10.3390/metabo12121273)
Supplement: Supplementary file 1 [file metabolites-12-01273-s001.zip › Figure S1.pdf]

(a)

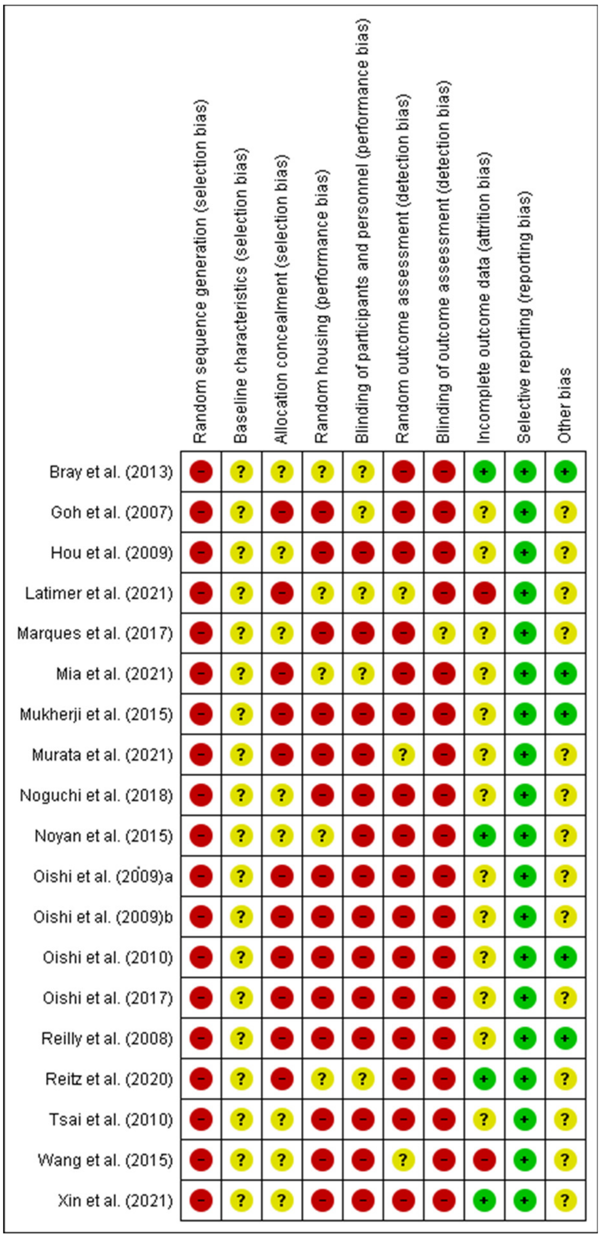


(b)

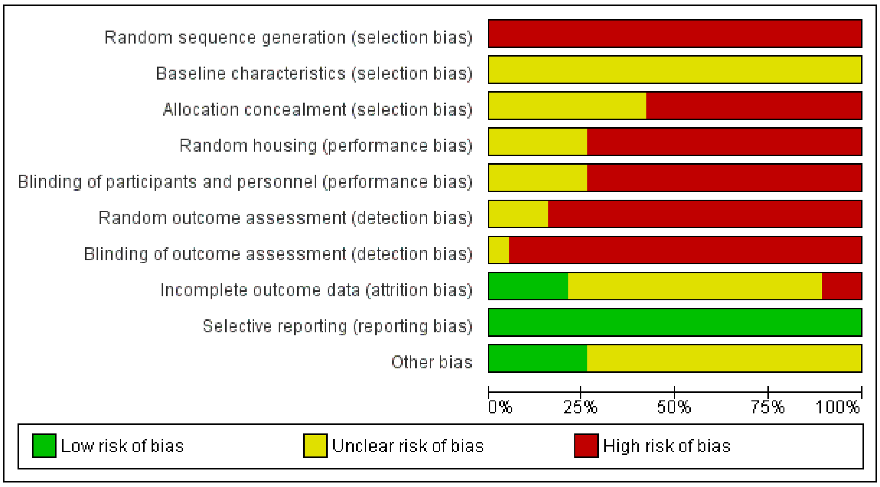


**Figure S1**. Result of the assessment of the risk of bias of the studies included in the systematic review. (a) summary of the risk of bias; (b) risk of bias graph.
