# Supplementary material for: The Effect of Diet on the Cardiac Circadian Clock in Mice: A Systematic Review
Source: Metabolites. 2022 Dec 15;12(12):1273. doi: 10.3390/metabo12121273 (PMC9786298; doi:10.3390/metabo12121273)
Supplement: Supplementary file 1 [file metabolites-12-01273-s001.zip › Table S1.pdf]

**SUPPLEMENTARY MATERIALS**

**Table S1.** Diets and its effects on the circadian clock.

| Dietary Interventions/Diet | Studies | Conclusion |
|---|---|---|
| **Restricted feeding** | 18–23 | Light phase: desynchronization between metabolically active tissues;<br>Dark phase: increased caloric intake, reduced energy expenditure, and dependence on fatty acid oxidation;<br>RF: Modulator of peripheral circadian oscillators, its effect on circadian rhythms is sex-dependent. |
| **High-fat diet** | 24,26–31 | Affected the peripheral circadian clocks;<br>RF with HFD in the dark phase: restores changes in lipid metabolism and cardiac remodeling;<br>The end of the active phase: myocardial steatosis, increased triglycerides synthesis;<br>Absent model CLOCK: Protective effect for cardiovascular diseases;<br>During pregnancy and lactation: affect the expression of clock genes, metabolism genes, inflammatory pathways;<br>RF with HFD: effect of endogenous insulin dependent in the feeding cycle on the regulation of peripheral clocks. |
| **Ketogenic diet** | 25,32 | Phase advance on clock genes, hypoglycemia, increased FFA and ketone body levels. |
| **Others diets** | 33–36 | Time of day of BCAA intake influences cardiac parameters;<br>Fiber dietary fiber and acetate act as a ZT;<br>Biotin-rich diet induced protein biotinylation;<br>Condition of hypophosphatemia increased levels of clock genes. |

Legend: RF, restricted feeding; HFD, high-fat diet; FFA, free fatty acid; BCAA, branched-chain amino acids; ZT, zeitgeber time.
